# Capacity of Arctic fjord sediments to degrade carbohydrates from permafrost active layer

**DOI:** 10.1128/spectrum.00456-26

**Published:** 2026-05-13

**Authors:** Chukwufumnanya Y. Abuah, Katie Sipes, Joy Buongiorno, Andrew D. Steen, James A. Bradley, Donato Giovannelli, Andrey Abramov, Samantha L. Peters, Richard J. Giannone, Robert L. Hettich, Renxing Liang, Julia Boike, Tatiana A. Vishnivetskaya, Karen G. Lloyd

**Affiliations:** 1 Department of Earth Sciences, University of Southern California 5116, Los Angeles, California, USA; 2 Department of Microbiology, University of Tennessee 4292, Knoxville, Tennessee, USA; 3 Department of Biological Sciences, University of Southern California 5116, Los Angeles, California, USA; 4 CNRS, IRD, MIO, Université de Toulon, Aix Marseille University, Marseille, France; 5 School of Biological and Behavioural Sciences, Queen Mary University of London, London, United Kingdom; 6 University of Naples “Federico II” Naples, Naples, Italy; 7 Institute of Physicochemical and Biological Problems of Soil Science, Pushchino, Russia; 8 Biosciences Division, Oak Ridge National Laboratory 6146, Oak Ridge, Tennessee, USA; 9 China University of Geosciences 12564, Wuhan, China; 10 Permafrost Research Section, Alfred Wegener Institute Helmholtz Centre for Polar and Marine Research, Potsdam, Germany; 11 Geography Department, Humboldt University Berlin, Berlin, Germany; Connecticut Agricultural Experiment Station, New Haven, Connecticut, USA

**Keywords:** microbial communities, metagenomics, permafrost, marine sediments, metatranscriptomics, fjords, CAZymes, climate

## Abstract

The degradation of organic matter (OM) by microorganisms in thawing permafrost produces greenhouse gases. Terrestrial OM is transported into fjords through hydrological runoff, but it is unclear whether the microbial mechanisms of OM degradation on land persist after soils enter marine environments, which differ greatly in conditions and microbial communities. This question is particularly relevant for low-OM soils, which dominate Arctic landscapes and are more exposed to oxidants. Here, we compared OM-degrading capacity in permafrost-affected active layer soils and adjacent fjord sediments from Kongsfjorden, Svalbard, focusing on carbohydrate-active enzymes (CAZymes), which target some of the most abundant types of organic matter in soils. Using multi-omics approaches—metagenomics, metagenome-assembled genomes (MAGs), metabolomics, metatranscriptomics, and metaproteomics—we examined CAZyme presence, distribution, and activity. Despite environmental differences, both soils and sediments harbored diverse glycoside hydrolases and polysaccharide lyases, most of which showed evidence of activity. Verrucomicrobia expressed the highest number of CAZyme transcripts, indicating that they dominated active carbohydrate degradation in fjord sediments, while Acidobacteria and Actinobacteria were more active in soils. Notably, CAZymes in fjord sediments targeted primarily soil-derived OM, and the proportions of enzymes degrading terrestrial OM, marine OM, and microbial necromass—remnants of dead microbial cells were similar across both environments. These results suggest that microbial communities in both soils and fjord sediments are equipped to degrade carbohydrates, and that burial of terrestrial-derived OM in fjord sediments may not protect it from microbial breakdown under Arctic warming.

**IMPORTANCE:**

Permafrost thaw may be a critical climate feedback because microbial degradation of organic matter (OM) can release greenhouse gases. While fjords serve as major carbon burial sites, our results show that burial of terrestrial-derived OM in these sediments does not ensure protection from microbial degradation. Microbial communities in both active layer soils and fjord sediments harbor a broad arsenal of carbohydrate-active enzymes, with evidence of activity across diverse taxa. This functional continuity indicates that once terrestrial material is washed into fjords, it remains vulnerable to microbial breakdown despite different environmental conditions. Understanding these cross-system continuities in microbial function is essential for predicting the fate of OM in a rapidly warming Arctic and highlights the importance of including fjord sediments in global carbon cycle models.

## INTRODUCTION

Permafrost soils are the largest terrestrial reservoir of organic carbon, storing approximately 1,400 Gt C—nearly four times the carbon currently in the atmosphere (1, 2). Climate change is driving unprecedented permafrost thaw, exposing this vast organic matter (OM) pool to microbial decomposition and transporting it, either dissolved or particulate, into rivers and fjords (3–5).

These processes have profound implications for the global carbon cycle, since the microbial mineralization of newly released OM can generate CO₂ or CH₄, exacerbating climate warming (2, 6, 7). The fate of permafrost-derived OM depends not only on the amount transported but on its chemical composition. Terrestrial OM contains structurally diverse carbohydrates such as cellulose, hemicellulose, and lignin-associated polymers, whereas fjord sediments also receive marine-derived polysaccharides and accumulate microbial necromass. These substrates differ in accessibility, redox sensitivity, and enzymatic requirements, making enzyme-substrate pairing a key determinant of whether OM is preserved or remineralized.

Although studies of microbial OM degradation tend to focus on areas with abundant OM (8–11), most of the land area of Arctic permafrost is comprised of mineral soils, with soil organic carbon (SOC) densities less than 50 kg SOC m^−2^ (1). Since OM in mineral soils is less dense, it may be more exposed to oxidants such as oxygen, iron, and nitrogen, as well as microbial enzymes.

While the vulnerability of Arctic OM to microbial degradation is increasingly recognized (12), less is known about its fate once it reaches coastal marine systems. Fjords, in particular, are significant repositories of terrestrial OM, receiving up to 17% of global terrestrial OM runoff and acting as long-term carbon sinks (13). For example, terrestrial OM accounts for 37%–78% of total OM in Kongsfjorden (14). Because little of this OM is degraded in the water column (15), fjord sediments serve as a crucial interface where OM is either buried and preserved or remineralized. Whether burial in fjord sediments protects terrestrial carbohydrates from microbial degradation remains unclear. (16). Microbial communities are key mediators of this processing, yet their structure, activity, and functional capacity across permafrost soils and fjord sediments remain poorly understood (17).

The Svalbard archipelago offers a compelling case study of these processes. Air temperatures have increased by approximately 1.5°C per decade since 1991 (18, 19), deepening the active layer and increasing runoff of permafrost-derived OM into fjords through enhanced glacier melt, rainfall, and extended streamflow (20, 21). Storm intensification further amplifies sediment and OM delivery to fjord basins (22). The declining seasonal snow cover on the active layer is expected to decrease the soil warming during the winter, thus shortening the available timeframe when microbial processing can occur (23). Together, these interacting changes make Svalbard’s fjords and their surrounding permafrost critical systems for studying carbon cycling under a warming Arctic.

Despite these dramatic changes, there remains a major knowledge gap in understanding how permafrost-derived OM is degraded as it transitions from land to marine sediments. It is also unclear whether microbial communities continue to remineralize soil OM after burial in fjords rich in oxidants such as iron. (24, 25). Therefore, this study asks: (i) What are the major microbial community differences between permafrost active layer soils and fjord sediments? and (ii) Do these environments differ in the types of carbohydrate substrates targeted for degradation, and does burial in fjord sediments alter the likelihood of terrestrial polymer remineralization?

To address these questions, we characterized microbial community structure in Svalbard permafrost active-layer soils and fjord sediments and assessed the genetic and expressed gene capacity for OM degradation, focusing on CAZymes. We focused on CAZymes because polymeric carbohydrates constitute a large fraction of terrestrial and marine OM and the CAZyme repertoires provide substrate-specific insight into which carbohydrate polymers are targeted, expressed, and potentially remineralized in each environment (26). We evaluated how carbohydrate-degrading processes could shape the likelihood of OM preservation versus remineralization during permafrost thaw and burial.

We integrated metagenomics, MAG reconstruction, metaproteomics (active layer), metatranscriptomics (fjord sediments), and metabolomics to resolve substrate-specific degradation potential and activity across environments. Although both the active layer and fjord sediments contain bacteria as well as eukaryotes such as fungi, we focused on bacteria due to their high abundance, their rapid responses to environmental change, and their well-characterized CAZyme repertoires (27–29). By resolving substrate-specific degradation potential and activity across environments, this integrative approach enables evaluation of whether transport to fjords promotes preservation or continued microbial processing of permafrost-derived carbohydrates under Arctic warming.

## MATERIALS AND METHODS

### Sediment sample collection

In March 2021, active-layer (AL) core samples were collected from frozen soils in Ny-Ålesund, Svalbard (Fig. S1). Two boreholes were drilled at the Bayelva site (78°92.1002 N, 11°83.2895 E), reaching depths of 115 and 45 cm, respectively. Cores were capped and stored in liners at frozen temperatures until subsectioning. For sectioning, cores were removed from the liners and sliced at 4-cm intervals using a sterile hammer and chisel.

Fjord sediments were collected in 2016 from four sites within two fjords: Van Keulenfjorden (VK) and Kongsfjorden (KF) (Fig. S1). Sediment cores were collected using a HAPS corer (30) and stored at approximately 5°C before processing 1–3 days later at the Ny-Ålesund Marine Laboratory. Cores were sterilely sectioned at 1-cm intervals down to ~20 cm and immediately frozen on dry ice. Samples remained frozen during transport to the University of Tennessee and were stored at –80°C until further processing.

### DNA extraction and sequencing

DNA was extracted from 0.5 g of soil sub-sampled at specified AL depth using the Qiagen DNA PowerSoil DNeasy DNA extraction kit. To achieve a total of 10 ng DNA yield for metagenomic sequencing, triplicate extractions were done for each sample and pooled. The resulting DNA from each sample was quantified using a Qubit fluorometer. DNA libraries were prepared using the SMARTer ThruPlex DNA-seq libraries (350 bp average fragment size) and sequenced on Illumina NovaSeq 6000 S4 (2 × 150 bp) at Science for Life Laboratory in Stockholm, Sweden. Details on each concentration can be found in reference 31.

The co-extraction of DNA and RNA from fjord sediments was performed using the Qiagen RNeasy Powersoil Kit for RNA with the DNA extraction accessory (Hilden, Germany) according to manufacturer’s instructions. RNA extracts were treated with DNAse I followed by sequencing of metatranscriptomic libraries with Illumina HiSeq, PE 2 × 250 bp. Individual 1 cm depth-resolved metatranscriptomic libraries were generated with RNA extracts from the upper 5 to 6 cm of sediment from all two fjord sites, for a total of 20 metatranscriptomes. Details regarding RNA and DNA extractions from fjord sediments can be found in reference 32.

### Bioinformatic analyses

Metagenome raw reads were trimmed for quality using Trimmomatic according to default settings (33), resulting trimmed reads were assembled separately using MetaSPAdes v.3.14.1 (34). Assembled contigs were annotated with Prokka v1.14.6 (35). Contigs were binned using three binning tools; CONCOCT (36), MetaBAT2 v2.15 (37), and MaxBin2 v.2.2.2 (38). DAS Tool v1.1.2 (39) was applied with default settings to each binned contig set to create medium-to-high-quality MAGS according to MIMAG criteria (40). The resulting bins were dereplicated using dRep through the metaWRAP (41) pipeline to remove redundant genomes. MAGs were taxonomically classified according to GTDB taxonomy using GTDB-tk-2.4.0 and GTDB release R214 (42). We used the CoverM module to calculate the length-weighted abundance of each MAG in each sample based on metagenomic read mapping (43). Metagenome assemblies were taxonomically classified using Kaiju (44). Although the original Kaiju web interface has been discontinued, the tool is currently available via the KBase platform. SignalP was run on CAZymes with Prodigal to determine extracellular CAZymes (45). To identify CAZyme enrichment across environments, we used the anvi-script-enrichment-stats tool from the Anvi’o 7.0 suite (46). This script calculates enrichment scores and statistical associations for annotated functions across groups of genomes or samples. We ran this analysis on CAZyme annotations from the fjord and active layer data sets to determine which CAZyme families were significantly enriched in either environment.

### Metabolic pathway analyses and data visualization

The Anvi-estimate-metabolism program was employed within Anvi’o v. 7.1 (46) using the default completeness cutoff and the –addcoverage option to predict the completeness of KEGG metabolic modules within individual MAGs. CAZymes analysis was performed on MAGs and assembled contigs using DRAM v1.2.4 on Kbase (47). Bar plots were generated in R using the ggplot2 package (48).

### Metabolomic and metaproteomic methods

Water-soluble metabolites were extracted from approximately 30 mg of permafrost material using a methanol:acetonitrile:water (2:2:1 v/v/v) solvent system containing 0.1 M formic acid. Untargeted metabolomic profiling was performed using ultra-high-performance liquid chromatography coupled to high-resolution Orbitrap mass spectrometry (UHPLC-HRMS) in negative-mode electrospray ionization. Chromatographic separation was achieved on a Synergi Hydro-RP column with a gradient elution program over 25 minutes. Mass spectra were acquired at 140,000 resolution across m/z ranges of 85–800 and 110–1,000. Metabolites were identified by exact mass (±5 ppm) and retention time comparison to an in-house library of 279 authenticated standards. Data processing was performed using the Metabolomic Analysis and Visualization Engine (MAVEN) for peak alignment and metabolite identification.

Proteins were extracted from 10 g of freeze-dried, sifted soil using alkaline lysis (0.1 M NaOH), followed by adjustment to 4% SDS and sonication. Following reduction with dithiothreitol and alkylation with iodoacetamide, proteins were purified using the protein aggregation capture (PAC) method with hydrophobic magnetic beads to remove humic substances. Proteins were digested with trypsin and analyzed by automated 1D LC-MS/MS using a Vanquish UHPLC system coupled to a QExactive-Plus mass spectrometer. Peptides were separated on a 75-µm inner diameter C18 nanospray column over a 190-min gradient. Database searches were conducted using Peaks Studio 11 against custom borehole-specific protein databases constructed from metagenomic contigs, with a 1% peptide-level false discovery rate threshold applied. Detailed protocols are provided in supplemental methods.

## RESULTS

### Microbial community composition across permafrost active layer and fjord environments through metagenomics

**Fig 1:**
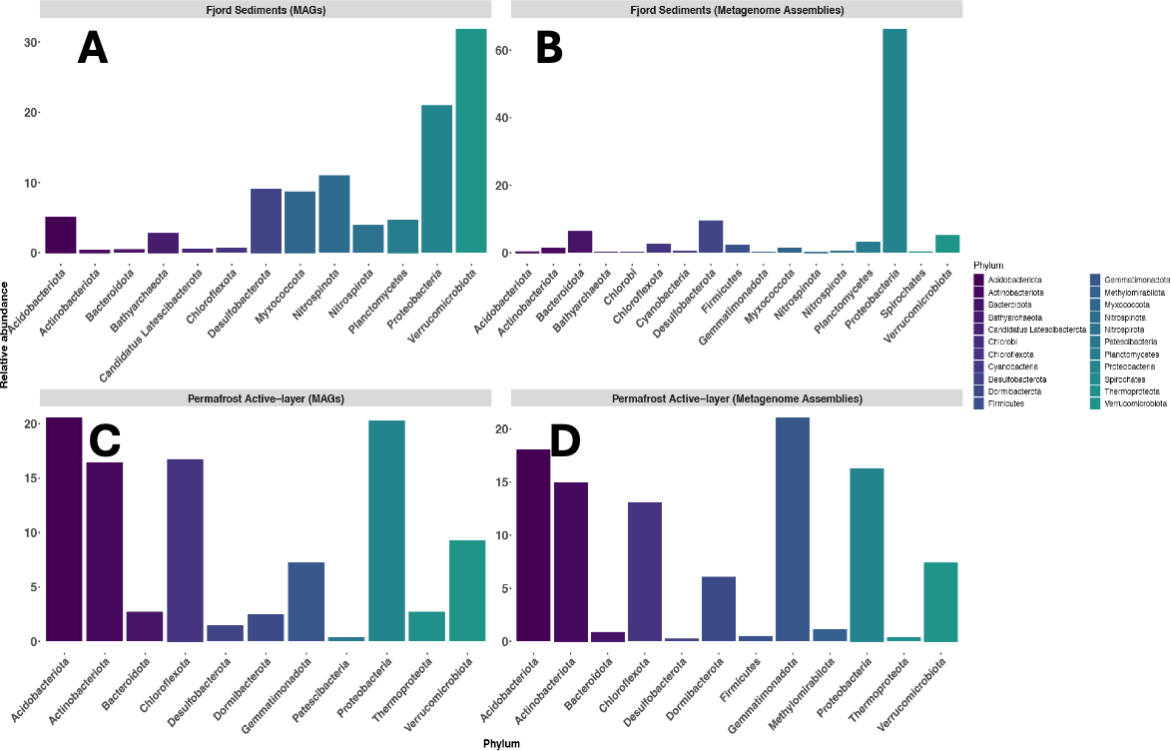
Taxonomic composition of fjord sediment and permafrost active-layer (AL) communities either based on metagenome-assembled genomes (MAGs) for (**A**) fjord sediments and (**C)** AL soils or based on metagenome assemblies for (**B**) fjord sediments and (**D**) AL soils. Panels **A** and **C** show the binned portion of the community (MAGs), whereas panels **B** and **D** show the full taxonomic profile based on unbinned metagenome assemblies.

In the fjord sediments, metagenomic binning resulted in a total of 49 medium- to high-quality metagenome-assembled genomes (40), ranging in completeness from 51% to 98%, while contamination ranged from 0% to 9.52% (Table S1). With the exception of one MAG that was identified as an archaeon, all MAGs were identified as bacteria. Fourteen MAGs were affiliated with the phylum Pseudomonadota (Proteobacteria), and all MAGs but one belonged to the class Gammaproteobacteria; the exception was classified as an Alphaproteobacterium. Nine MAGs were members of the Verrucomicrobia phylum, and seven MAGs belonged to Desulfobacterota. Four MAGs were representatives of Planctomycetes, two MAGs were Chloroflexota, and two MAGs were Bacteroidota. In addition, we had one MAG representative of each of the following phyla: Acidobacteria, Actinobacteria, Gemmatimonadota, Myxococcota, and Thermoproteota Bathyarchaeota within the Archaea (Fig. 1).

In the AL data set, metagenomes were assembled and binned separately, and the resulting medium- to high-quality MAGs were dereplicated across samples to yield 44 distinct genomes. (Table S2). This comprised 16 high-quality MAGs and 28 medium-quality MAGs with completeness ranging from 51.32% to 98% and contamination ranging from 0 to 8.7%. Acidobacteria and Actinobacteria have the highest representation with 10 and 8 MAGs, respectively. Seven MAGs were identified within the Chloroflexi phylum, and five MAGs within Verrucomicrobia. The Bacteroidota and Pseudomonadota (Proteobacteria) each have four MAGs, while Gemmatimonadota and Thermoproteota (Nitrosospharaceae) each have two MAGs. Desulfobacterota, Dormibacterota, and Patescibacteria are each represented by one MAG.

### Genomic potential for organic matter degradation in AL and fjords

**Fig 2:**
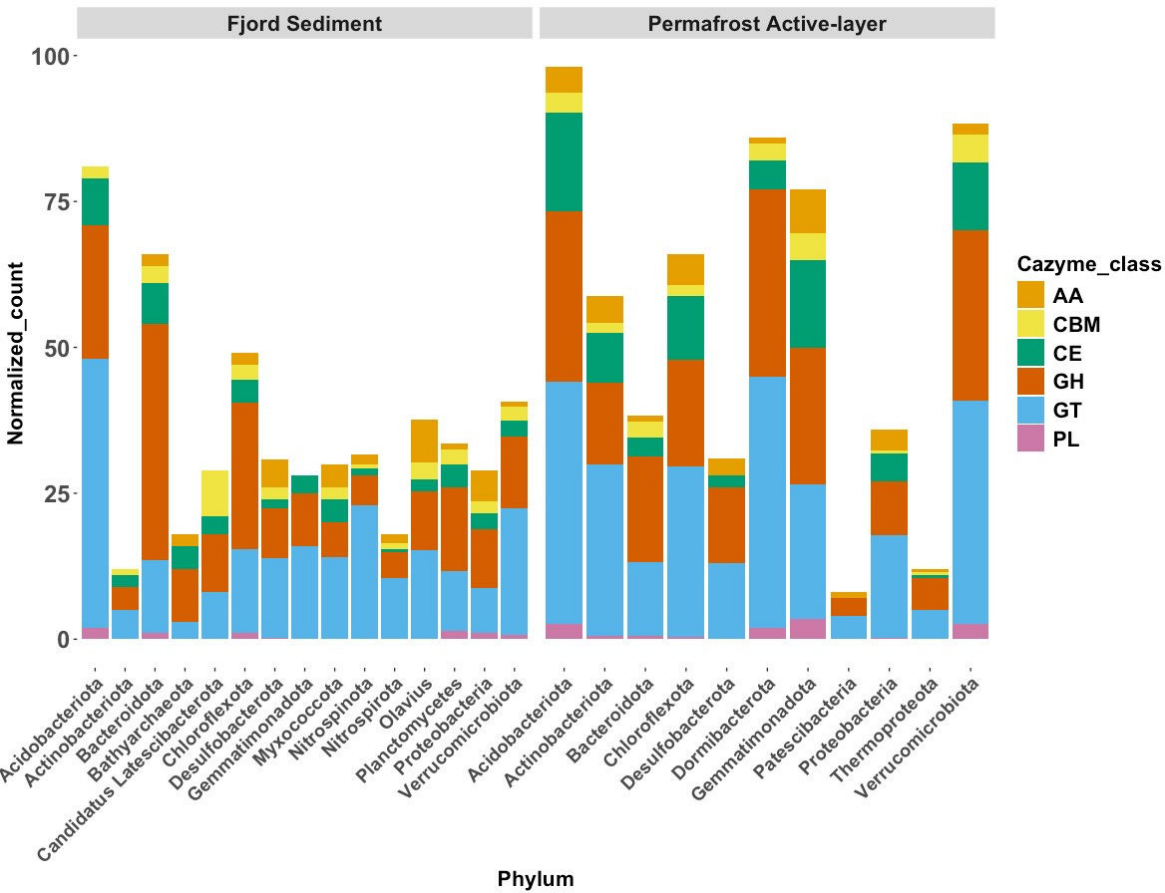
CAZymes in metagenomes. Stacked bar plot showing the total counts of CAZyme genes across all phyla in fjord sediments and the permafrost active layer (AL), normalized by the total number of MAGs within each phylum. Each segment within the stacked bars represents a distinct CAZyme class.

All MAGs were examined for their individual OM degradation potential based on CAZyme profiles. Across all MAGs, we identified 2,956 CAZyme hits in the AL and 1,762 in the fjord data set. Glycosyltransferases (GTs) were the most abundant class (43% AL; 40% fjord), followed by glycoside hydrolases (GHs; 30% AL; 34% fjord), carbohydrate esterases (CEs; 15% AL; 8.5% fjord), auxiliary activity enzymes (AAs; 6% AL; 8.5% fjord), carbohydrate-binding modules (CBMs; 3.7% AL; 6% fjord), and polysaccharide lyases (PLs; 1.9% in both, Fig. 2).

In total in the MAGs, we found 23 GH families unique to the fjord and 21 unique to the AL (Table S5). Unique CAZymes in fjord sediments primarily targeted marine-specific and sulfated polysaccharides, such as agarose, fucoidan, and glycosaminoglycans, reflecting adaptation to algal and detrital marine inputs (49). In contrast, the AL harbored CAZymes geared toward plant-derived polysaccharides, including cellulose, hemicellulose, and chitin, consistent with terrestrial OM breakdown (50).

The most abundant GH families were shared across both environments (GH109, GH13, GH2, GH23, GH3, and GH74), and target substrates like cellulose, peptidoglycan, and starch (Fig. S2). However, divergence emerged beyond the top six: fjord sediments were enriched in GH106, GH29, GH171, and GH1, while the AL showed higher abundances of GH57, GH5, GH33, GH15, and GH77 (Fig. S3). The GH13 family, which targets starch, was not only among the most abundant GH families but also was strongly enriched in the active layer metagenomes. Additional CAZyme families enriched in the active layer included CBM32 and PL9, both associated with the degradation of pectin—a major structural component of plant cell walls. A complete list of enriched CAZymes is provided in Table 1.

**TABLE 1:** CAZyme families enriched in permafrost active layer soils relative to fjord sediments, along with their corresponding enrichment scores and adjusted *P*-values

CAZyme family	Enrichment score (In AL)	Adjusted *P*-value
CBM82 (β-glucans)	8.43	0.014
CBM48 (glycogen)	8.43	0.014
GH116 (β-glucosidases)	7.14	0.022
GH13_26 (Starch)	13.89	0.004
GH19 (chitin)	5.88	0.015
GH42 (lactose)	5.88	0.015
GH47 (mannan)	7.14	0.022
PL9 (pectin)	11.09	0.004
GT21 (β-glucans)	11.08	0.001
GT84 (glycosaminoglycan)	7.14	0.022
GT89 (glycan)	5.88	0.015

**Fig 3:**
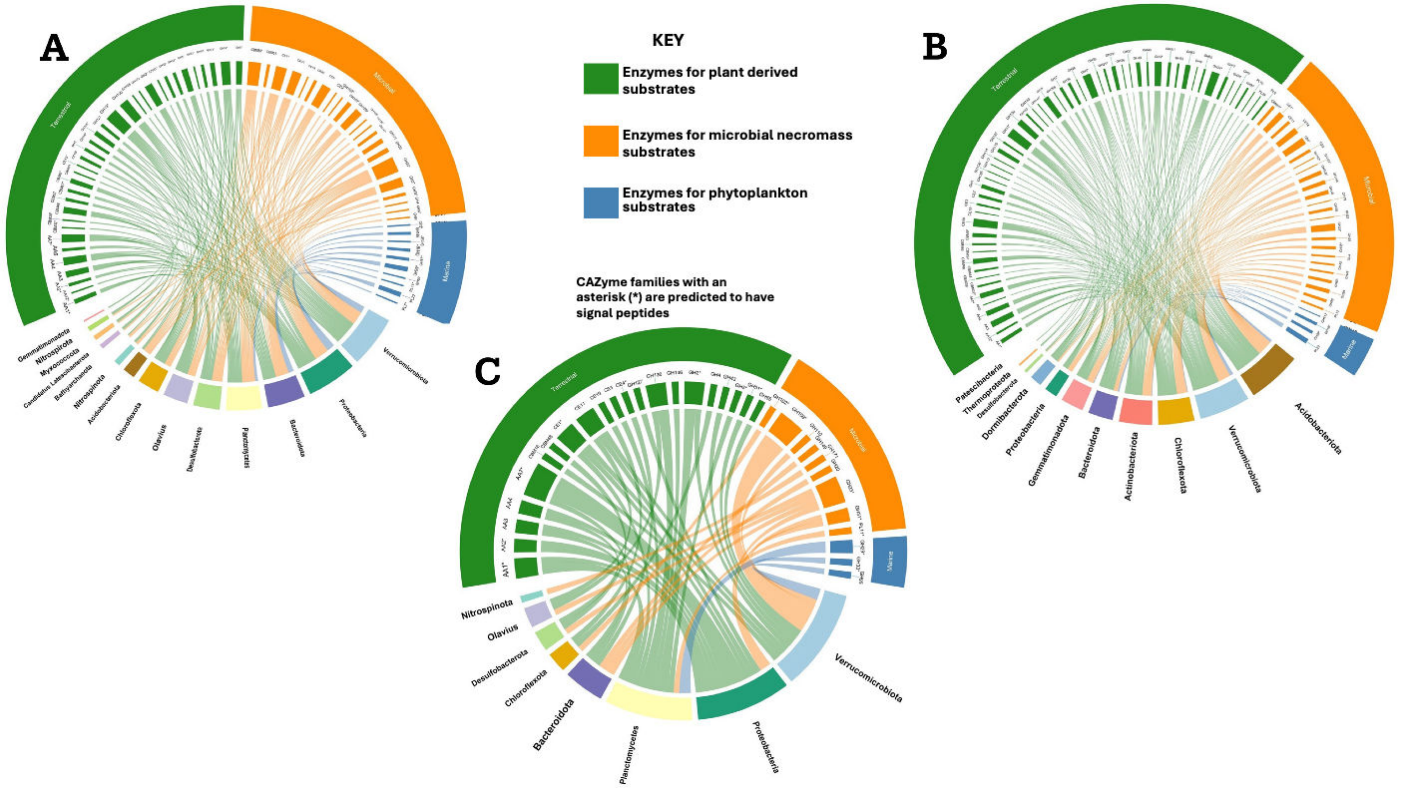
CAZyme distribution and activity across fjord and active layer microbial communities. Chord diagrams showing recruitment of metagenomic or metatranscriptomic reads to metagenome-assembled genomes (MAGs) and their associated carbohydrate-active enzyme (CAZyme) families. (**A**) Metagenomic recruitment to MAGs from fjord sediments. (**B**) Metagenomic recruitment to MAGs from active layer (AL) soils. (**C**) Metatranscriptomic recruitment to MAGs from fjord sediments. MAGs are grouped by phylum, and CAZyme families are grouped by predicted substrate origin: plant-derived (green), microbial necromass-derived (orange), and marine-derived (blue). CAZyme classes include glycoside hydrolases (GH), carbohydrate esterases (CE), auxiliary activities (AA), carbohydrate-binding modules (CBM), and polysaccharide lyases (PL). Asterisks (*) indicate families predicted to contain signal peptides.

Based on their predicted target substrates, CAZymes were grouped into three substrate categories: terrestrial (plant-derived), marine (algal-derived), and necromass (microbial cell wall components), following classifications established in previous studies (51). Some individual CAZymes may fall in multiple categories, but these classifications are useful for getting an initial estimate of the way carbohydrates are degraded in these ecosystems. This grouping of CAZyme families by substrate origin showed that, in the fjord metagenomes, 50% targeted terrestrial OM, 39% microbial necromass, and 9.2% marine-derived substrates. In the AL, the distribution was 64% terrestrial, 30% necromass, and 4.4% marine (Fig. 3).

### Carbon-degrading pathways in fjords confirmed by metatranscriptomics

In the fjord metatranscriptomes, we detected 84 CAZyme transcripts and, excluding the GTs, 55 CAZyme transcripts were as follows: 30 GHs, 14 AAs, 8 CEs, 2 CBMs, and 1 PL; GTs were excluded from downstream analysis as they are only involved in assembly and not degradation (28) (Fig. 3C). The most transcripts were derived from Verrucomicrobia (15 transcripts), followed by Proteobacteria (13 transcripts), Planctomycetes (11 transcripts), Bacteroidota (6 transcripts), Olavius (3 transcripts), Chloroflexota (3 transcripts), Desulfobacterota (3 transcripts), and Nitrospinota (1 transcript) (Fig. 3C). Highly transcribed CAZymes included AA7 (cellobiose dehydrogenase, five hits), GH109 (N-acetylgalactosaminidase, four hits), GH23 (peptidoglycan endo-β−1,4-N-acetylmuramidase, four hits), and a unique PL transcript from Planctomycetes. Grouping these transcripts by substrate origin revealed that the majority (63%) were associated with terrestrial OM, followed by 27% linked to necromass, and 6.9% targeting marine-derived substrates.

### Carbon-degrading pathways in AL confirmed by metaproteomics

**Fig 4:**
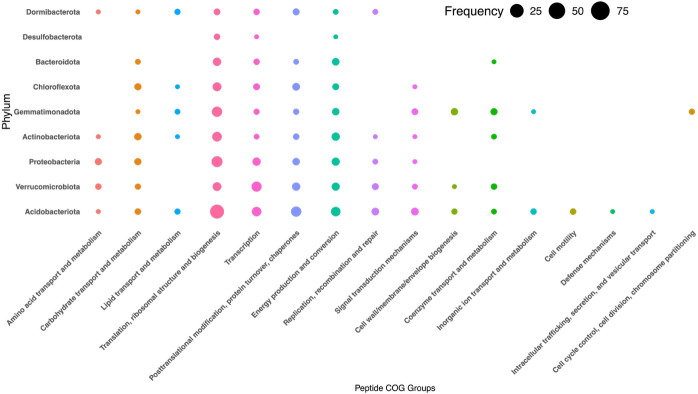
Total metaproteome of AL sites distributed across identified phyla and protein COG category. The area of each bubble corresponds to the summed chromatographic abundance of all peptides belonging to each COG annotation across all MAGs in that phylum.

Transcripts were not obtained in AL due to low RNA yield, so proteomics data were used for activity inference. In the AL, proteomic analyses confirmed active carbohydrate metabolism under low temperature conditions, with 184 peptides identified (Table S3). Carbohydrate-metabolism proteins accounted for 4.6% of total abundance, the most expressed being glyceraldehyde-3-phosphate dehydrogenase. Acidobacteria showed the highest peptidase activity (231 hits), followed by Verrucomicrobia (94 hits), Proteobacteria (89 hits), Gemmatimonadota (77 hits), Actinobacteria (52 hits), and Chloroflexota (51 hits) (Fig. 4) (Table S3). The most represented functional COG categories included energy production and conversion (97 hits), transcription (95 hits), and post-translational modification (81 hits).

### Metabolomic analyses of fjords and AL

**Fig 5:**
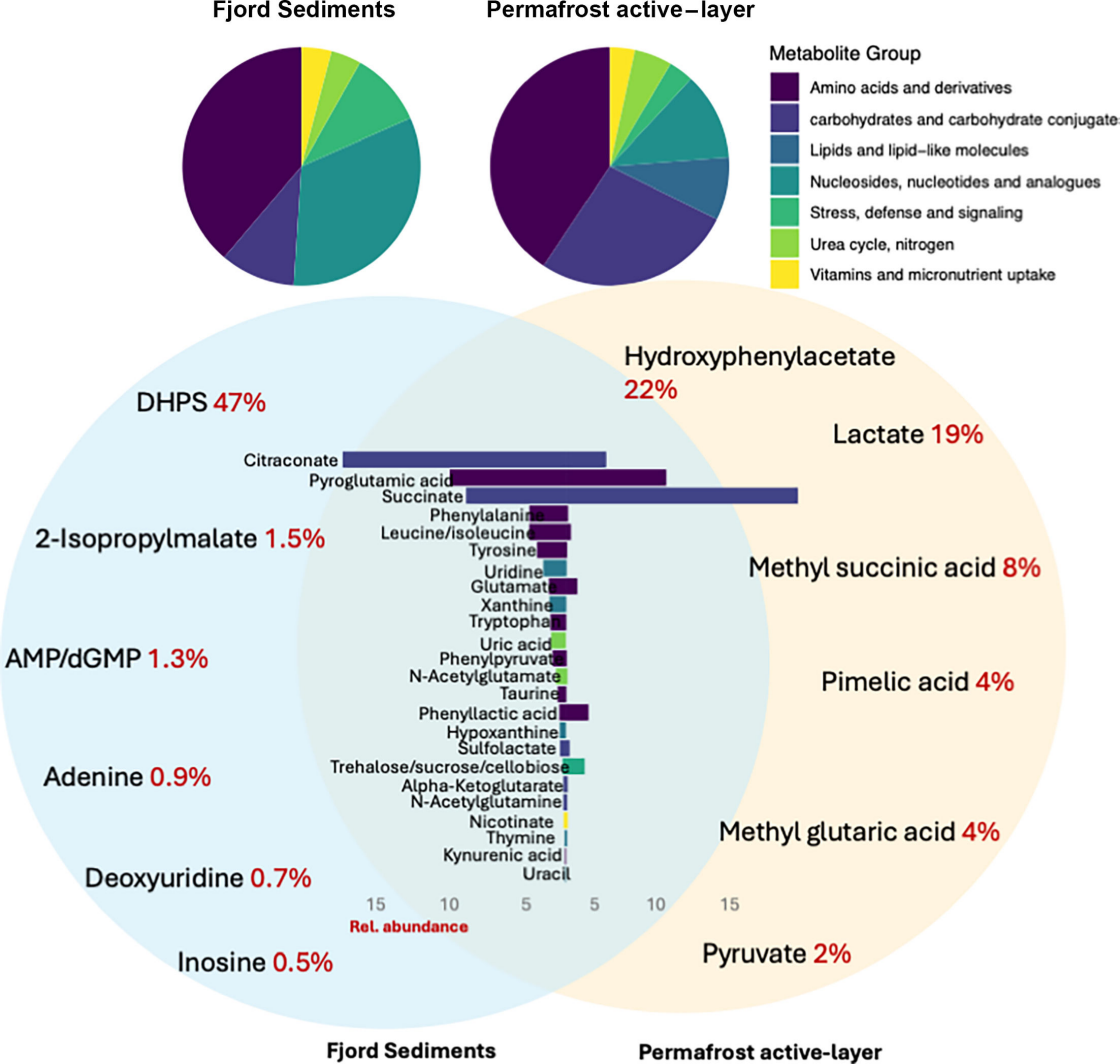
Fjord sediment and AL metametabolome. Pie charts displaying the relative abundance of all metabolites, categorized for fjord sediments and AL. A bar plot highlighting only the shared metabolites in the center of the Venn diagram, with their relative proportions in fjord sediments (left) and AL (right). Venn diagrams illustrate the six most abundant site-specific metabolites, with their relative abundance values indicated in red.

Metabolomic analysis identified a total of 49 compounds in the fjord and 61 compounds in the AL (Table S4; Fig. 5). Metabolites in these samples are interpreted to reflect a combination of intra- and extracellular components, and some of them have been found previously in subsurface samples (52, 53). In the fjord, the largest group of compounds (39%) is amino acids, such as tryptophan, tyrosine, methionine, and phenylalanine, or amino acid derivatives, such as kynurenic acid and acetyllysine. Nucleic acids and their derivatives constitute 33% of the metabolite pool, followed by metabolites related to stress, defense, and signaling (10%), energy metabolism (10%), vitamin and micronutrient uptake (4%), and urea/nitrogen cycling (4%). In the AL, the majority of the metabolites (38%) are amino acids and their derivatives. Nucleic acids and their derivatives constitute 10% of the metabolite pool. Lipids and lipid-like molecules constitute 10%, and carbohydrate metabolic pathways constitute 25% of the pool. A total of 38% of the metabolites found in the fjord are not found in the AL, and 35% of the metabolites from the AL were not found in the fjord. A majority of metabolites uniquely found in the active layer soils were sugars, while in the fjord, the unique metabolites were mostly nucleic acid constituents and amino acids (Fig. 5). The full list of metabolites can be found in Table S4.

## DISCUSSION

Combining metagenomics, metatranscriptomics, metaproteomics, and metabolomics of microbial populations in active layer (AL) soils above permafrost and neighboring fjord sediments in Svalbard (79°N) gives a nuanced view of what types of microbes and carbon degrading potential are shared between them. This is important because both of these environments are potentially important in degrading SOC to greenhouse gases, such as carbon dioxide and methane, as this SOC thaws and is washed into fjords. However, the taxa and carbon-degrading capabilities are expected to differ greatly between these two environments, since AL is terrestrial and undergoes seasonal freezing and thawing with exposure to air, while the fjord sediment is submerged year-round under marine-influenced fjord waters and never freezes. Despite these environmental contrasts, our results show that degradation potential for carbohydrates from terrestrial and marine environments persists across systems, indicating that burial in fjord sediments does not inherently protect terrestrial carbon from microbial processing.

Although many phyla are shared between the two environments, phyla, such as Dormibacteriota and Patescibacteria, are exclusive to AL, while Myxococcota and Bathyarchaeota appear only in fjord sediments (Fig. 1), suggesting ecological specialization in how OM is processed before and after transport to marine systems. The good agreement between MAGs and total metagenome assemblies (Fig. 1) suggests that the differences in taxa between these two environments are not due to differences in bias in MAG binning.

Within the AL, Acidobacteria is the most abundant phylum in both the metagenome and the metaproteome (Fig. 1 and 4), consistent with its known role in degrading sugars and proteins in permafrost environment (54, 55). While all phyla appeared in the metaproteome, only Acidobacteria, Gemmatimonadota, and Verrucomicrobia expressed proteins linked to cell division and membrane biogenesis, suggesting these groups may be actively growing during frozen winter conditions (Fig. 4). Other phyla expressed proteins involved in core functions like energy production, transcription, and translation. Nearly all phyla except Desulfobacteria expressed proteins involved in protein, carbohydrate, or lipid metabolism, agreeing with metabolomic evidence showing abundant sugars, amino acids, and lipid-like compounds in the AL (Fig. 4 and 5).

In contrast, fjord sediments are dominated by Proteobacteria in both abundance (metagenomes) and activity (metatranscriptomes) (Fig. 1), followed by Verrucomicrobia. Despite its high abundance, Desulfobacteria showed limited CAZyme transcription (Fig. 3C), indicating minimal contribution to OM degradation at the time of sampling. Although Proteobacteria had the total highest abundance and recruited transcripts, Verrucomicrobiota specifically showed the highest number of CAZyme transcripts, followed by Planctomycetes (Fig. 3C). Despite its lower relative abundance, Planctomycetes contributed substantially to transcribed CAZymes, suggesting that carbohydrate breakdown in fjord sediments may be driven by lower abundance taxa. Such taxa often occupy narrow ecological niches, possessing highly efficient enzymatic repertoires that allow them to exploit specific substrates or persist under resource-limited conditions (56). Moreover, transcriptional dominance of these groups may reflect temporal activation or opportunistic substrate utilization rather than steady-state abundance (56). Actinobacteria, although prominent in AL, were largely absent from the fjord community, consistent with observations from other studies in Kongsfjorden (57).

### Terrestrial substrates

As expected from AL, since it is terrestrial, the most abundant and diverse CAZymes there target terrestrial substrates, with evidence from metagenomes, metatranscriptomes, metaproteomes, and metabolomes (Fig. 3). However, we found that this is also the case in fjord sediments, in spite of their heavy marine influence. In fact, most (>80%) GH groups are shared between AL and fjord sediments. Even CAZymes that differ between AL and fjord sediments often target similar substrates, suggesting that burial into fjord sediments does not eliminate the microbial potential to degrade terrestrial carbon. For example, GH93 and GH8, found only in the AL, target arabinoxylan and xylan, but fjord sediments contain GH158 and GH10, which act on the same compounds. The abundance of transcripts for these enzymes in fjord sediments suggests that terrestrial substrate degradation remains ecologically important in this environment, even though it is not terrestrial.

### Lignin: distinct microbial strategies reflect geochemical contexts

Lignin is the most abundant aromatic polymer in plant biomass and is also a component of coal which is abundant in Svalbard bedrock (58–60). It likely enters fjord sediments via ancient petrogenic inputs (61), since present-day plant biomass is low. In the fjord metagenomes, lignin-degrading auxiliary activity (AA) genes—redox enzymes that degrade complex aromatics (62) —are concentrated in Proteobacteria and Desulfobacteria. These AA genes constitute over 35% of terrestrial CAZymes transcribed, with Proteobacteria alone contributing more than half. In addition, Proteobacteria and Planctomycetes transcribe non-CAZyme enzymes like hydroxyaminophenol mutase and 4-hydroxyphenylacetate decarboxylase, further supporting aromatic compound degradation (63, 64).

In contrast, AL showed a broader distribution of AA genes across phyla, with Acidobacteria harboring the most diverse and abundant set. Metabolite evidence supports this activity with vanillin and hydroxybenzoate, both lignin breakdown products detected only in AL sediments (Table S3). These patterns suggest that lignin degradation occurs in both systems but is likely carried out by different microbial taxa. In the AL, diverse groups, including Acidobacteria and Actinobacteria, may contribute to lignin breakdown. However, because the active layer remains frozen for much of the year, degradation may be incomplete, leading to the accumulation of intermediates. In contrast, the fjord’s consistently wet, unfrozen sediments and high turnover rates are likely driven by iron- and sulfur-driven redox processes (25). These conditions, fueled by glacial inputs and seawater diffusion, may provide the energy needed for more efficient degradation of lignin and its intermediates, indicating that lignin buried in fjord sediments is not necessarily protected from microbial processing.

### Cellulose: conserved function across divergent communities

Cellulose, the most abundant plant polymer (65), is, like lignin, degraded in both environments by different taxa. GH2 and GH5 families, present in both systems, target cellulose (28). GH2 is present in the fjord sediment metagenomes within Verrucomicrobia, Planctomycetes, Gemmatimonadota, and Chloroflexota. In the AL, however, GH2 is found only in Acidobacteria, Bacteroidota, and Dormibacterota. This suggests that cellulose may be degraded in both AL and fjord sediments, but by different microbial phyla. In the fjord, GH2 was actively transcribed by Planctomycetes and Verrucomicrobia, indicating that cellulose; aother major terrestrial substrate can be degraded by these phyla upon reaching the fjord environment.

### Xylan: active degradation in the AL, modified pathways in the fjord

Xylan, a major hemicellulose in terrestrial plant biomass (66), represents an important carbon source in permafrost ecosystems (67). Multi-omic evidence points to highly efficient xylan degradation in the AL, especially by Actinobacteria, which produce more carbohydrate-degrading proteins than any other group. They actively express pathways for xylose uptake and metabolism, consistent with other enrichment studies (68, 69). This robust activity likely achieves near-complete xylan breakdown before it can be exported to fjord sediments.

In the fjord sediment, although xylan-targeting GH10 and GH158 genes are present in MAGs, they are not transcribed. This suggests that the xylan in the fjord may not be bioavailable, likely due to structural features such as higher substitution or algal-associated modifications that differ from terrestrial xylan (70).

The absence of detectable transcription of xylan-degrading enzymes suggests that xylan in fjord sediments may not be actively undergoing microbial degradation. Similar patterns, where xylans persist for millennia due to incomplete microbial remineralization, have been documented in other marine sediments (70–72), supporting the idea that structurally altered or recalcitrant forms (including those from algal sources) may accumulate in fjord sediments despite microbial potential for degradation.

Together, these substrate-specific patterns reveal that while certain polymers (e.g., xylan) may be extensively degraded in the active layer prior to export, others (e.g., lignin and cellulose) remain accessible to fjord microbial communities. This indicates that transport to marine sediments does not uniformly protect terrestrial carbohydrates; instead, degradation potential is substrate-dependent and shaped by redox and community composition differences between systems.

### Evidence for OM degradation in frozen conditions

Even though our samples were collected at the end of winter, when the soils had been frozen for several months, there is evidence for ongoing or residual metabolic activity through metaproteomics and metabolomics. Core TCA cycle enzymes; pyruvate dehydrogenase, malate dehydrogenase, citrate synthase, and isocitrate dehydrogenase (Table S3) were detected in the proteomes alongside their corresponding metabolites (citrate, malate, and α-ketoglutarate), many of which were exclusive to the active layer (Fig. 4 and 5, and Table S4). The co-occurrence of these enzymes with their metabolic intermediates suggests maintenance of basal metabolic functions under subzero conditions, consistent with reports that cold-adapted microbes can sustain slow but measurable respiration and protein turnover even in frozen soils (73, 74). This finding demonstrates that degradation processes begin before thaw-driven export, meaning that permafrost-derived carbon may already be partially processed before entering fjord systems. Nevertheless, we cannot entirely exclude the possibility that some protein or transcript signatures represent preserved “molecular fossils” of earlier summer activity stabilized by freezing. Both mechanisms; low-level winter activity and biochemical preservation likely contribute to the observed signal.

### Marine-derived substrates

We identified fewer CAZymes targeting marine substrates such as fucoidan, alginate, carrageenan, and other sulfated-polysaccharide–degrading enzymes in both the AL and fjord sediment metagenomes (Fig. 3). This was true for both gene and transcript abundances (Fig. 3). Despite the fjord’s marine-influenced setting, low transcription of marine-degrading enzymes suggests these substrates may be less abundant, likely due to limited algal productivity caused by high turbidity (15).

Some marine-targeting CAZymes (such as GH50, PL6, and PL7), which degrade alginate and ulvans, were found exclusively in fjord metagenomes. A few were also detected in the AL, likely reflecting cross-environmental exchange. This exchange may enable the microbial community to degrade marine polysaccharides, even in terrestrial settings. Although these CAZymes are encoded by multiple phyla, their expression in fjord sediments was limited to Planctomycetes and Verrucomicrobia, suggesting these taxa are particularly adapted to processing algal-derived polysaccharides in marine sediments. This pattern aligns with broader observations of Planctomycetes and Verrucomicrobia as key players in macroalgal polysaccharide degradation across marine systems (49, 51), enabling them to capitalize on seasonal algal inputs while other microbes focus on terrestrial organic matter.

Despite the lower transcription of CAZymes targeting marine substrates compared to terrestrial ones, metabolite data suggest that marine polysaccharide degradation is still occurring. Specifically, DHPS (2,3-dihydroxypropane-1-sulfonate), a known breakdown product of sulfonated marine polysaccharides like carrageenan and agar (75), accounts for nearly 50% of all detected metabolites in the fjord yet is absent from the AL (Fig. 5). Together, these results indicate that while the fjord microbial community retains the capacity to degrade marine substrates, transcriptional activity overwhelmingly favors terrestrial organic matter, indicating that land-derived OM delivered to fjord sediments remains vulnerable to degradation.

### Microbial necromass substrates

Our multi-omics data suggest that the breakdown of microbial necromass is a key metabolic strategy in both fjord sediments and the AL. The most abundant CAZyme for degrading microbial necromass across all phyla in both systems is GH23, a peptidoglycan-degrading enzyme (76). GH23 is widespread across MAGs, frequently occurs in multiple copies, and is actively transcribed by Proteobacteria, Desulfobacterota, Olavius, and Bacteroidota in fjord sediments, showing its ecological significance.

Other abundant families, such as GH109 and GH31, which target glycan-rich necromass components (28) are also consistently present in both metagenomes and transcriptomes. Even Nitrospinota, a phylum that only had one transcript, expresses GH171, an enzyme that degrades N-acetyl muramic acid, another key cell wall component (77). These patterns support the idea that necromass degradation is widespread across taxa, which supports the suggestion that necromass oxidation can supply up to 13% of microbial energy needs in shallow sediments under both oxic and anoxic conditions (78). Mineral adsorption may further restrict access to other organic substrates, making cell wall polymers like peptidoglycan prime targets for secreted CAZymes (78). Supporting this, a global multi-omics study showed that peptidoglycan-degrading proteins are most abundant in marine metaproteomes, suggesting that necromass recycling is a dominant process in fjord sediments (79).

In the AL, GH23 is also the most abundant necromass-degrading CAZyme, highlighting its role in peptidoglycan turnover beyond the fjord. GH109 is particularly enriched in Verrucomicrobia, which have the highest gene copy numbers. Of the six statistically enriched CAZyme families in the AL, three—GH19 (chitinase), GH42 (β-galactosidase), and GH47 (mannosidase)—are linked to microbial and fungal necromass degradation (Table 1). These enzymes occur exclusively in Acidobacteria and Verrucomicrobia, implicating them as major contributors to necromass turnover. This is further supported by their high peptide counts: 232 hits for Acidobacteria and 94 for Verrucomicrobia.

Overall, the widespread presence and expression of CAZymes targeting peptidoglycan, chitin, β-glucans, and β-galactosides across environments and phyla suggests microbial necromass degradation as a shared and essential strategy for survival in these sediment ecosystems.

In direct response to our initial questions, we find that although microbial community composition differs substantially between permafrost active layer soils and fjord sediments, functional potential for terrestrial carbohydrate degradation is largely conserved across systems. However, substrate-specific differences, particularly in hemicellulose and aromatic processing, indicate that degradation pathways are shaped by environmental context and redox conditions. Together, these results demonstrate that microbial community shifts during land-to-sea transport do not eliminate carbohydrate-degrading capacity but rather reorganize it among taxa.

### Conclusions

Although microbial communities and their carbon-degrading enzymes differ significantly between soils and fjord sediments, both environments exhibit a strong capacity to degrade organic matter (OM) originating from land. Fjords are widely recognized as efficient carbon sinks, with multiple studies documenting substantial burial and long-term preservation of terrestrial OM (13, 80). However, our findings suggest that microbial communities in fjord sediments retain the enzymatic potential and, in some cases, the active expression and direct metabolic evidence required to continue breaking down OM even after burial. This suggests a more nuanced view of fjord carbon cycling: while a large portion of OM is likely stabilized and sequestered, a fraction remains biologically available and subject to remineralization within the upper sediment layers. In Svalbard fjords, this active degradation zone may extend up to the first meter, supported by labile anaerobic electron acceptors such as ferric iron and sulfate.

As warming and increased precipitation intensify permafrost thaw and sediment delivery to fjords, our findings suggest that increased delivery of permafrost-derived carbohydrates to fjords under Arctic warming is likely to stimulate continued microbial processing rather than encourage stabilization, possibly enhancing CO₂ production and drawing long-term carbon sequestration into question. Our results emphasize the importance of considering microbial activity when evaluating the long-term fate of terrestrial carbon in marine sediments.

## Data Availability

Metagenomic sequences for the fjord sediments can be accessed in the NCBI SRA under BioSamples SAMN10372305–SAMN10372307 under BioProject PRJNA493859. Metagenome assemblies for fjord assemblies can be found at NCBI under accession numbers JANIFD000000000, JANIFE000000000, and JANIFF000000000. Metatranscriptomic reads can also be found under the same BioProject as metagenomic sequences. The metagenomic libraries for the active layer soils can be found under NCBI Metagenomic BioProject SAMN41246650.

## SUPPLEMENTAL MATERIAL

Supplemental figures spectrum.00456-26-s0001.docx Fig. S1 to S3 and supplemental methods.

Supplemental tables spectrum.00456-26-s0002.xlsx Tables S1 to S5.

ASM does not own the copyrights to Supplemental Material that may be linked to, or accessed through, an article. The authors have granted ASM a non-exclusive, world-wide license to publish the Supplemental Material files. Please contact the corresponding author directly for reuse.
