# Supplementary material for: Capacity of Arctic fjord sediments to degrade carbohydrates from permafrost active layer
Source: Microbiol Spectr. 2026 May 13;14(6):e00456-26. doi: 10.1128/spectrum.00456-26 (PMC13228077; doi:10.1128/spectrum.00456-26)
Supplement: Supplemental figures — Fig. S1 to S3 and supplemental methods. [file spectrum.00456-26-s0001.docx]

**Supplemental Tables and Figures**

**Genetic and transcriptomic capacity for microbial degradation of soil-derived carbohydrates in the permafrost active layer and neighboring Arctic fjord sediments**

Authors: Chukwufumnanya Y. Abuah^1^, Katie Sipes^2^, Joy Buongiorno^2^, Andrew D. Steen^3^, James A. Bradley^4^, Donato Giovannelli^5^, Andrey Abramov^6^, Samantha L. Peters^7^, Richard J. Giannone^7^, Robert L. Hettich^7^, Renxing Liang^8^, Julia Boike^9,10^, Tatiana A. Vishnivetskaya^2^, Karen G. Lloyd^1*^

^1^Department of Earth Sciences, University of Southern California, Los Angeles, CA

^2^Department of Microbiology, University of Tennessee, Knoxville, TN

^3^Department of Biology, University of Southern California, Los Angeles, CA

^4^Aix Marseille University, Université de Toulon, CNRS, IRD, MIO, Marseille, France

^4^School of Biological and Behavioural Sciences, Queen Mary University of London, London, U

^5^University of Naples “Federico II” Naples, Italy

^6^Institute of Physicochemical and Biological Problems of Soil Science, Pushchino, Russia

^7^Biosciences Division, Oak Ridge National Laboratory, Oak Ridge, TN

^9^China University of Geosciences, Wuhan

^9^Alfred Wegener Institute, Potsdam, Germany

^10^Humboldt University Berlin, Geography Department, Unter den Linden 6, 10099 Berlin, Germany.

**Fig. S1. Map and core collection sites.**
(A) Map showing the locations where active layer soil cores and fjord sediment cores were collected.
(B) Photograph of an unsectioned active layer soil core.
(C) Photograph of an unsectioned fjord sediment core.


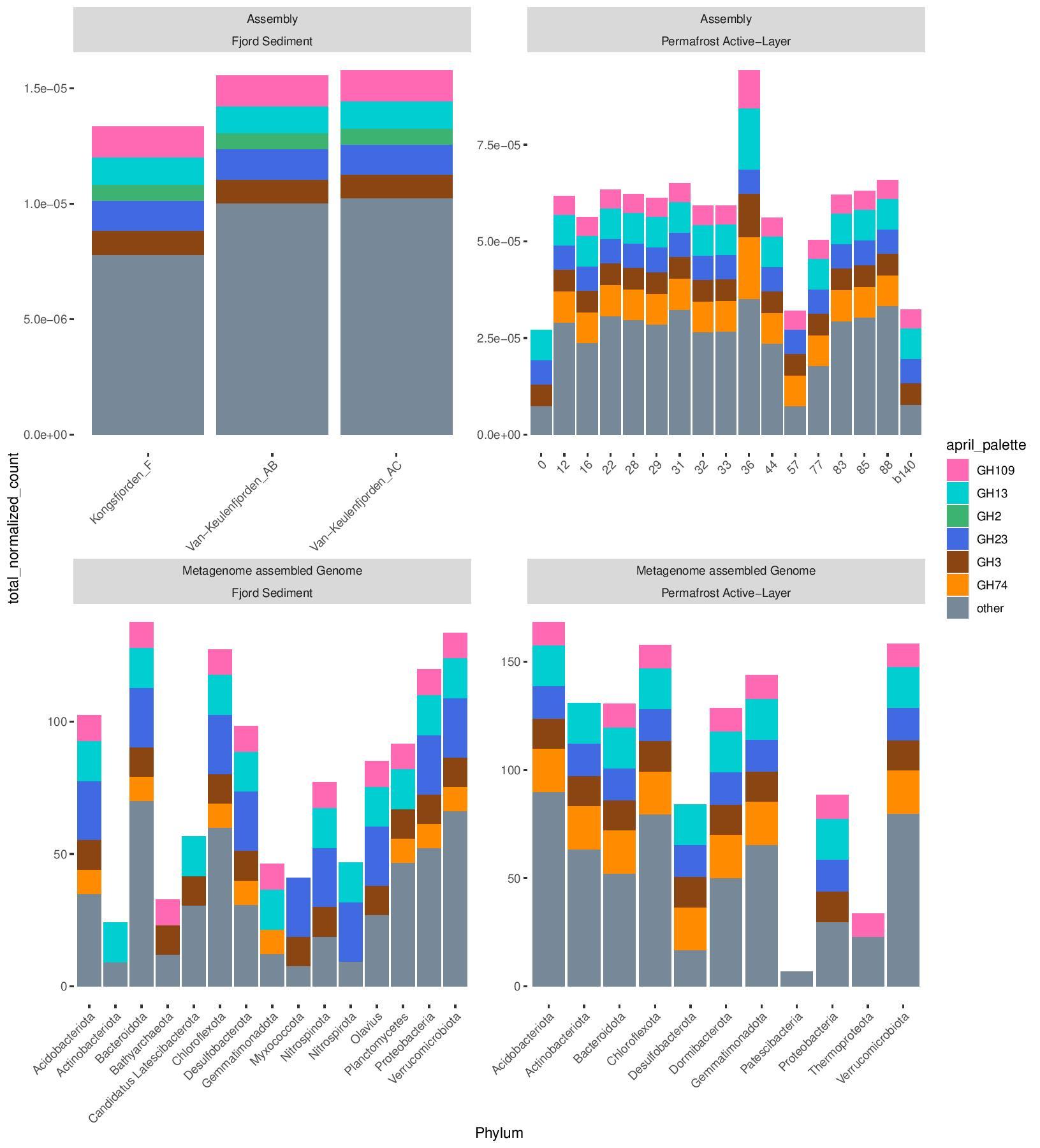


**Figure S2**: Stacked bar plots showing most abundant (top 6) glycoside hydrolase families by count across all phyla and assemblies in fjord sediments and AL


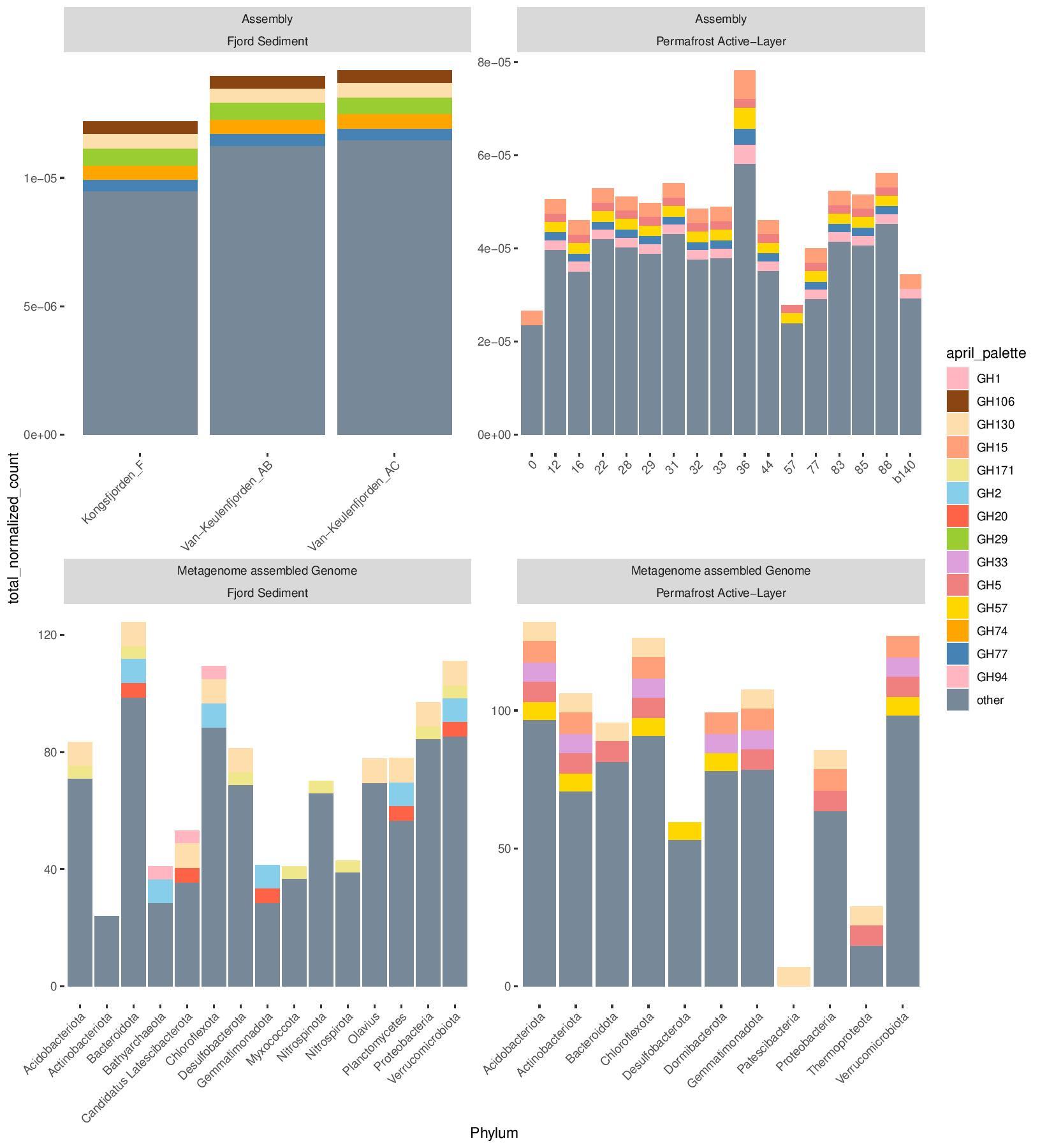


**Figure S3**: Stacked bar plots showing most abundant (top 6-10) glycoside hydrolase families by count across all phyla and assemblies in fjord sediments and AL

**Supplemental methods -**

**Metabolomics Extraction and UHPLC-HRMS Analysis**

Approximately 30 mg of permafrost material was subjected to untargeted metabolomics

analysis at the Biological and Small Molecule Mass Spectrometry Core at the University of

Tennessee, Knoxville (RRID: SCR_021368). Water-soluble metabolites were extracted using an

established protocol optimized for polar metabolite profiling. 2 HPLC-grade solvents (Fisher

Scientific, Hampton, NH) were used throughout. The extraction solvent consisted of methanol,

acetonitrile, and water containing 0.1 M formic acid, mixed in a 2:2:1 (v/v/v) ratio. Each sample

was extracted with 1.3 mL of this solvent at −20 °C for 20 minutes. Samples were centrifuged at

15,000 RPM for 5 minutes, and the supernatant was collected. An additional 200 µL of

extraction solvent was added to the remaining pellet and re-extracted under the same

conditions. Supernatants from both steps were combined and dried under a stream of high-

purity nitrogen gas. Dried samples were resuspended in 300 µL of water prior to LC-MS analysis.

Metabolomic analysis was conducted using an ultra-high-performance liquid chromatography

system coupled to a high-resolution Orbitrap mass spectrometer (UHPLC-HRMS), following a

previously validated method for water-soluble metabolites. 1 Chromatographic separation was

performed on a Synergi Hydro-RP column (2.5 μm, 100 Å, 100 × 2.0 mm; Phenomenex,

Torrance, CA), maintained at 25 °C and operated using an Ultimate 3000 UHPLC pump (Dionex,

Sunnyvale, CA). Samples were held at 4 °C in the autosampler during the course of analysis. The

mobile phase consisted of solvent A (97:3 [v/v] water:methanol with 10 mM tributylamine and

15 mM acetic acid) and solvent B (100% methanol). The gradient elution began with 100%

solvent A, transitioned to 80% A and 20% B by 5 minutes, 45% A and 55% B by 13 minutes, and

reached 5% A and 95% B by 15.5 minutes. The gradient then returned to 100% A by 19 minutes

and was held until the end of the 25-minute run. The flow rate was maintained at 200 μL/min.

The LC effluent was introduced into an Exactive™ Plus Orbitrap mass spectrometer (Thermo

Scientific, Waltham, MA) via negative-mode electrospray ionization (ESI) using a 0.1 mm

internal diameter fused silica capillary. Instrument settings included a spray voltage of 3.0 kV,

sheath gas flow of 10 (arbitrary units), capillary temperature of 320 °C, and an AGC target of 3 ×

10⁶ ions. Mass spectra were acquired at a resolution of 140,000, with full-scan acquisition from

m/z 85–800 from 0 to 9 minutes and m/z 110–1000 from 9 to 25 minutes.

Data Processing

Raw spectral files generated by Thermo’s Xcalibur 4.0 software, were converted to the

open source mzML format by utilizing the msConvert package from ProteoWizard. 3 After

conversion, the mzML files were uploaded to Metabolomic Analysis and Visualization Engine

(MAVEN). This program was used for peak alignment and retention time correction.

Metabolites were manually identified by exact mass (± 5 ppm) and retention times, which were

compared to an in-house library of 279 metabolite standards.

**LC-MS/MS-based Metaproteomics**

*Sample preparation.*. Soil samples were freeze-dried and sifted to remove large debris prior to cellular lysis and protein extraction.

12 mL of 0.1M NaOH was added to 10 g of dry, sifted soil and samples were incubated at room

temperature for 30 minutes, shaking at 600 rpm. After incubation, samples were centrifuged at 1000 x g for 30 minutes to pellet soil debris. The supernatant was transferred to a new tube and adjusted to 4% (wt:wt) sodium dodecyl sulfate (SDS) before sonication (Branson sonifier) at 40% amplitude with two seconds on/off intervals for two minutes. Samples were centrifuged at 4,500 x g to pellet debris and the supernatant was transferred to new tubes for further processing. Proteins were reduced with 10 mM dithiothreitol (heating at 90°C for 10 minutes) and alkylated with 30 mM iodoacetamide (incubated in the dark for 20 minutes). Extracted proteins were further cleaned and prepared for proteolytic digestion via the protein aggregation capture (PAC) method (ref) that was adapted to extract proteins and remove humic substances from a complex soil matrix. In brief, 300 μg of hydrophobic magnetic beads (1 micron, SpeedBead Magnetic Carboxylate; GE Healthcare UK) and acetonitrile (ACN) were added for a final concentration of 70% ACN to induce protein aggregation on the beads. Samples were incubated for 20 minutes at room temperature before centrifugation at 4,500 x g to pellet beads. The supernatant was removed and pelleted beads were subjected to three rounds of washes with 2mL of ACN, followed by 2mL of 70% ethanol, and 2mL of 1% formic acid. The final 1% formic acid wash buffer was removed from tubes and the beads were resuspended in 4% (wt:wt) sodium deoxycholate/100mM ammonium bicarbonate for in-solution proteolytic digestion. Samples were subjected to sonication at 20% amplitude with two seconds on/off intervals for 1 minute to fully resuspend beads. 5 ug of proteomics-grade trypsin (Pierce) was added to samples and incubated at room temperature, shaking, for overnight digestion. An additional 5ug of trypsin was added for a second 3-hour digestion period at 37°C, shaking at 600 rpm. Samples were acidified with 1% formic acid and centrifuged at 4,500 x g for 10 minutes. The supernatant was transferred to a new tube and three rounds of ethyl acetate clean-up

were performed to remove any residual SDC. Peptides were filtered on a 10kDa MWCO filter, frozen, and freeze-dried. Samples were resuspended in 40uL solvent A (95% H2O, 5% acetonitrile, 0.1% formic acid).

*LC-MS/MS Measurements.* LC-MS/MS 20uL of peptide solution (1/2 of extraction volume) were analyzed by automated 1D LC-MS/MS analysis using a Vanquish ultra-HPLC (UHPLC) system plumbed directly in-line with a QExactive- Plus mass spectrometer (Thermo Scientific). A 100  µm inner diameter trapping column (packed to 10 cm with 5 µm Kinetex C18 reverse-phase resin (Phenomenex)) was coupled to an in-house-pulled 75 µm inner diameter nanospray emitter (packed to 15 cm with 1.7 µm Kinetex C18 reverse-phase resin (Phenomenex)). Peptides were loaded, desalted, and separated by uHPLC under the following conditions: sample injection followed by 100% solvent A (95% H2O, 5% acetonitrile, 0.1% formic acid) from 0 to 30 min to load and desalt, a linear gradient from 0 to 30% solvent B (70% acetonitrile, 30% water, 0.1% formic acid) from 30 to 220 min for separation, and 100% solvent A from 220 to 240 min for column re-equilibration. Eluting peptides were analyzed with the following MS settings: data-dependent acquisition, top-10 method; mass range 300–1500 m/z; MS and MS/MS resolution 70 and 15 K, respectively; MS/MS loop count 20; isolation window 1.8 m/z; charge state exclusion of unassigned, +1,+6–8 charges.

*LC-MS/MS Data analysis.*Database searches were conducted with Peaks Studio 11 against custom borehole-specific protein databases constructed from all contigs in any sample collected from the borehole, as well as common mass spectrometry contaminants. Sequences were clustered at 100% amino acid sequence identity (exact amino acid length) prior to database searches. For all database searches, the parent and fragment ion mass error tolerances were set to ± 10 ppm and ± 0.02 Da, respectively. Accepted modifications included a fixed modification of carbamidomethylation (+57.02) of cysteine residues and variable modifications of oxidation (+15.99) of methionine and deamidation of (+0.98) of asparagine and glutamine residues, with a maximum of three variable modifications allowed per peptide. Semi-tryptic digestion of peptides was allowed, and a 1% peptide-level FDR threshold was applied.
